# A universal high voltage SF6 circuit breaker for HVDC and HVAC systems

**DOI:** 10.1038/s41598-024-82786-w

**Published:** 2025-01-07

**Authors:** Fady Wadie, Tamer Eliyan

**Affiliations:** 1 Mechatronics and Robotics Engineering Department, Faculty of Engineering, Egyptian Russian University, Badr City, Egypt; 2 Department of Electrical Engineering, Faculty of Engineering at Shoubra, Benha University, Cairo, 11629 Egypt; 3 Department of Electrical Power and Machines Engineering, Alshorouk Academy, The Higher Institute of Engineering at El-Shorouk City, Cairo, 11837 Egypt

**Keywords:** Transmission line, HVDC, HVAC, Circuit breaker, SF6, Energy science and technology, Engineering

## Abstract

High voltage alternating current (HVAC) and high voltage direct current (HVDC) transmission systems have acted as the pillars for the transmission field in power networks. Thus, two types of circuit breakers (CBs) existed; HVDC-CB and HVAC-CB. This imposed a burden on manufacturers by requiring them to set up a separate production line for each type of them, increasing production time and overall cost. This paper proposes a solution for this problem by combing both types into a single universal frame termed as UHAD-CB that could be utilized in both systems. Achieving this aim would reduce the manufacturing obstacles and benefit the entire industry. The proposed UHAD-CB was based on the structure of HVDC-CB which utilizes SF6 interrupter equipped with shunt L-C branch. The UHAD-CB is tested in both HVDC and HVAC systems and the results proved its reliability in both systems. The evaluation analysis for the performance of UHAD-CB showed its ability to reduce the transient recovery voltage by 28% in comparison to conventional HVAC-CB. This increase in performance rates allowed the usage of SF6 interrupter with lower cooling power than that for HVAC-CB which compensates for the added cost for L-C branch. In addition, the overall reduction in cost from the manufacturing point of view would add to that benefit. Finally, the optimum L and C values were found to be in range of 0.1 mH and 50 µF respectively for the selected systems.

## Introduction

High voltage transmission systems have grown to be the connecting nervous system for modern power networks. Their function of transmitting bulk power across wide distances have defined their operating level and type either DC or AC. Hence, two main types of high voltage transmission have been available high voltage AC (HVAC) and high voltage DC (HVDC) transmission with each of them utilized for different purposes^1,2^. For HVDC systems, they are mainly involved in transmitting bulk power internationally across nations. This role specifically fits HVDC systems for their inherited ability of interconnecting unsynchronized networks^3–9^. Meanwhile, there is still other applications where HVAC transmission is utilized. That include domestic transmission for extremely long distances over geographically challenging terrain^10–12^. Hence, it could be summarized that the utilization of either HVAC or HVDC is indispensable to power systems. For such reason, there is a requirement for protection equipment including high voltage circuit breakers for each of them that fits the requirements of each system.

For HVAC circuit breakers (CBs), the use of SF_6_ gas within switchgear in most of the high voltage substations have shown its suitability for operation in HVAC systems^13^. However, for HVDC systems, the CBs would require additional modifications to perform their switching function^14,15^. Theses modification are included to handle the absence of a natural current zero crossing by artificially injecting one^16,17^. Thus the interrupting SF6 element is equipped with a shunt L-C commutation circuit to provide an oscillatory current and a zero-crossing allowing current interruption^18–22^. An R-C shunt branch and a metal oxide variastor (MOV) are used to control the rate of rise of recovery voltage and absorb the excess energy following the arc interruption process respectively^23–28^.

The previous differences in the design of HVDC-CB and HVAC-CB have hindered their manufacturing process. As each of them required a dedication production line, assembly process and suitable supply chain. This problem is the main topic addressed in this work. The common element in both HVDC-CB and HVAC-CB is the same which is the SF_6_ interrupter. That opens a possibility proposing a universal SF_6_ based circuit breaker that is applicable in both HVDC and HVAC systems. That is the main aim of this paper. Achieving this aim could revolutionize the manufacturing process for the high voltage circuit breaking technology allowing less burden for assembly lines, higher rate of production, lower manufacturing costs and lower per-unit costs for utilities and consumers. Thus, the entire power system community would benefit from the proposed concept.

The proposed concept for *U*niversal *H*V*D*C/HV*A*C *CB* or UHDA-CB is based on key stages, firstly proposing key concepts, modeling and testing the proposed model. The modeling for SF6 interrupter is available either as physical or black box arc models. Black box models are commonly used among researchers in response to the high complexity of the physical model. The black box models the non-linear arc conductance^29–32^. Within black box models, Cassie and Mayr dynamic arc equations are the most fundamentally used models^19,20^. Mayr model is used in the modeling of SF6 interrupter of the UHDA-CB in this paper. The main contributions for this paper are summarized as follows:

1. Proposing a concept for a universal frame for high voltage CBs that is usable in both HVDC and HVAC systems.
2. Studying the performance of the proposed UHAD-CB in HVDC and HVAC systems.
3. Investigating the impact of different design elements (L, R and C) upon the performance of UHAD-CB.
4. The main contribution for UHAD-CB proposed in this work is its providing a path way that could be followed by other researchers to achieve a universal CB for HVDC and HVAC systems. Such achievement would revolutionize the industry allowing manufacturers to focus on a single production line for UHAD-CB rather than two lines for HVDC-CB and HVAC-CB. This would reduce supply burden, speed-up the manufacturing process, cut-down the production cost and reduce the final cost for CBs. Reaching a winning situation for all parties within the power system operational network.

The rest of this paper is organized as follows. Section 2 presents the proposed concept for UHAD-CB, its fundamental idea and structure. Section 3 proceeds with modeling process for the UHAD-CB and testing systems. Section 4 puts the UHAD-CB into test by placing it into a real transmission line that is under-construction in Egypt using ATP/EMTP. Simulation results are presented in the same section and discussed in the following Sect. 5. Finally, conclusions are drawn in Sect. 6.

## Proposed Concept for UHAD-CB

The concept of UHAD-CB is based on its main function which is to safely operate in HVDC or HVAC. For HVAC systems, the used interrupters do not require specific modifications. Meanwhile for HVDC systems, the interrupters are modified to include shunt L-C branch. This added branch is used to compensate for the absence of natural zero-crossing for currents as mentioned in the Introduction section. Another additional branches are also included; a damping resistive branch and MOV. It is clear that the difference between HVDC and HVAC CBs is in the additional shunt branches for HVDC-CBs. These shunt branches are considered indispensable for applicability of CB in HVDC systems because of their role in injecting an artificial zero-crossing. Therefore, for the UHAD-CB concept to be applicable to both systems, it had to combine these shunt branches in its design.

**Fig. 1:**
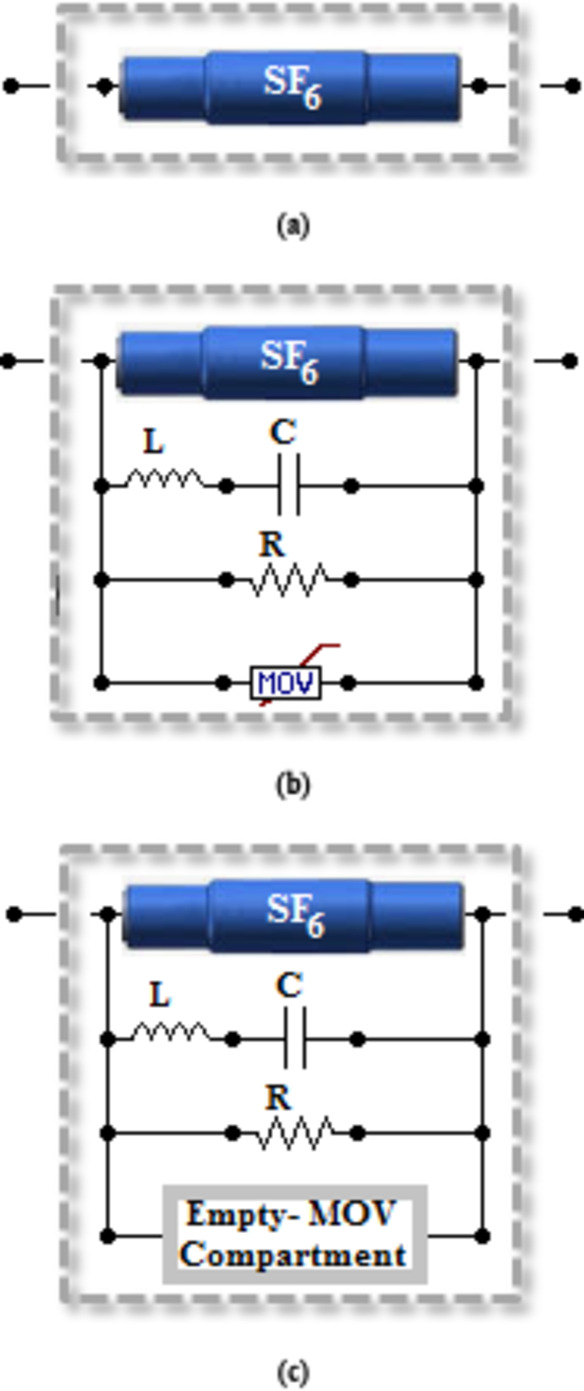
Frame diagrams for (**a**) HVAC-CB (**b**) HVDC-CB (**c**) UHAD-CB.

The previous argument articulates the basic topology of the UHAD-CB and its main components. The UHAD-CB would include the SF6 interrupter as the main interrupting element. Shunt LC and resistive branches would also be included. An empty compartment is provided that is designed to fit MOV in case its needed. When the UHAD-CB is used in HVDC system, an MOV is inserted into that compartment, otherwise it is kept empty. The previous design would allow the UHAD-CB to be applicable to both HVDC and HVAC systems. The design elements for UHAD-CB, HVAC-CB and HVDC-CB are shown in Fig. 1.

The performance of the UHAD-CB in HVDC systems is efficient as its design is based on HVDC-CB design. The remaining challenge would be testing its performance in HVAC systems. Hence, the first task in the simulation process would be evaluating the performance in UHAD-CB in HVAC system. Secondly, the additional branches added to the CB might enhance the performance but would increase the per-unit cost. This could be compensated by reducing one of the parameters of the SF6 interrupter. For example, if HVAC-CB uses an SF6 interrupter with a cooling power of 100 MW, the UHAD-CB could use an interrupter with less cooling power. Hence, the increase in cost for added branches is compensated by using less powerful interrupter. From technical point of view, the performance of the UHAD-CB should remain acceptable in both cases. The previous point would be tested in simulation process. Finally, the most suitable values for L and C within both systems will investigated. Hence, the main aims for the simulation process would be:

1. Test the performance of UHAD-CB in HVAC system to be acceptable with less powerful SF6 interrupter.
2. Compare the performance of UHAD-CB with HVAC-CB equipped with more powerful interrupter.
3. Evaluate the performance of UHAD-CB in HVDC systems.
4. Define the suitable values for L and C components for HVDC and HVAC systems.

## Modeling of the testing systems

Two systems were selected for testing the UHAD-CB, HVAC and HVDC transmission systems. Each of the two systems and their modeling are presented in this section. Finally, the modeling of the UHAD-CB is presented.

### HVAC transmission system

A 500-kV, 124 km transmission line located in El-Kuorimate – Cairo, Egypt was selected as the HVAC testing system. The data of the system are provided in Table 1.^33^ The line was modelled using LCC JMarti frequency-dependent-model available on ATP/EMTP.

**Table 1:** Data of HVAC Transmission System.

Positive & negative sequence impedance per phase in ohm	3.307 + j14.053
Zero sequence impedance per phase in ohm	10.75 + j45.67
No. of sub-conductors per phase	3
No. of ground wires	2
Diameter of sub-conductor in mm	30.6
Vertical height of conductor at mid-span in meter	9
Vertical height of ground wire at mid-span in meter	21
Diameter of ground wire in mm	11.02
DC resistance of conductor in ohm per km	0.0133
DC resistance of ground wire in ohm per km	0.35
Ground tower rod length in meter	1.5
Ground tower rod radius in cm	1.25

### HVDC Transmission System

**Fig. 2:**
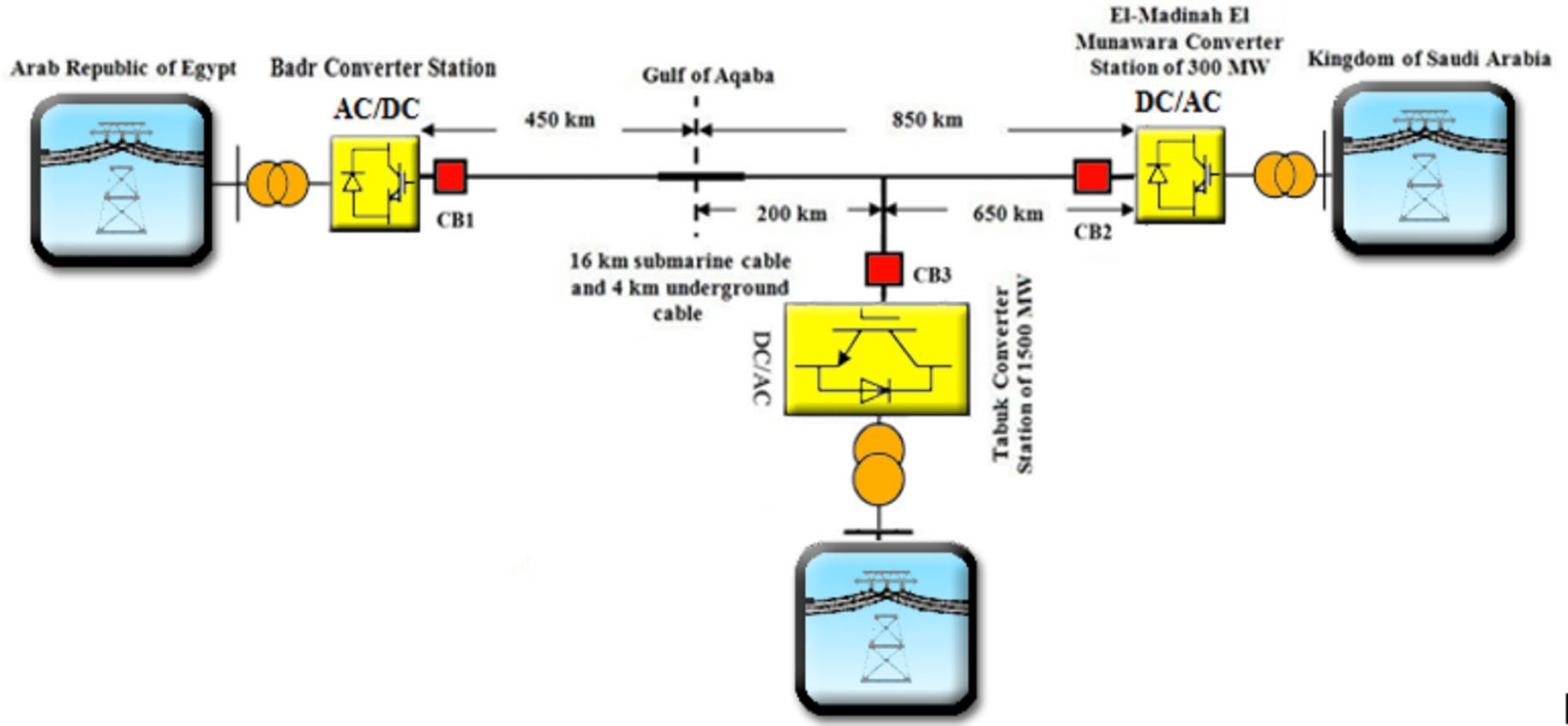
The HVDC transmission line between Egypt and the Kingdom of Saudi Arabia.

A real HVDC transmission line project that is currently under construction to connect Egypt and the Kingdom of Saudi Arabia has been selected as the HVDC testing system. The project includes 1,300 km transmission line with two 500 kV AC/DC substations and a linking station as shown in Fig. 2^20^. This work focuses on a 500 kV, 450 km portion of the line that is located in Egypt and connects Badr substation to Elnabaq switching station. A 500 kV source is used to model the selected system as a power supply providing it to 650 Ω load. The line is modeled as a 24.5 Ω resistor and 45 mH inductor connecting the source and the load^20^.

### Modeling of the SF6 interrupter

The UHAD-CB model relies on the SF6 interrupter as its core element. As mentioned in the introduction section, Mayr’s black box model is used for this purpose. Mayr’s modeled has a significant ability for performing dynamic analysis for the state of the arc that could be used to determine the CB’s capability of interrupting the arc^18^. The model computes the value of the conductance of arc in dynamic bases to define the transitional effect from the closed state to open state of the CB. During that transition, four stages are encountered these are closed breaker stage, arcing stage, arc extinguishing stage and open stage^34,35^. The closed and opened stages are represented by resistances of values 1 µΩ and a suitably high value of MΩ respectively. The arcing stage and the extinguishing stage are modeled using a variable resistor whose value is based on the conductance computed from the following mathematical deduction^34^: The consideration of environmental factors are incorporated in indirect way in the model and its results showed to acceptable among researchers in^18,20,22 – 23^. With practical testing done to validate the results of Mayr’s model in^23^.

- The energy stored within the arc $\:Q\left(t\right)\:$will be equal to difference between arc heating power $\:{(P}_{H})$ and cooling power due to energy dissipated from the arc $\:{(P}_{o})$. The energy stored within the arc during the interruption process $\:Q\left(t\right)\:$is given in (1). Both of the previous powers are affected the dimensional properties of the arc including its diameter and length. Hence, the following includes the impact of the arc’s length and diameter^34,35^.

1 $$\:\frac{dQ\left(t\right)}{dt}={P}_{H}-\:{P}_{o}$$

- The stored energy $\:Q\left(t\right)\:$ is used to compute the conductance of the arc $\:{g}_{m}\left(t\right)$ as given in (2) where τ is the arc time constant.

2 $$\:{g}_{m}\left(t\right)=K\:\frac{Q\left(t\right)}{{P}_{o}\:\tau\:}$$

Where K is the proportionality constant.

- The first equation in (1) could be rewritten in terms of the arc conductance as in (3).

3 $$\:\frac{dQ\left(t\right)}{d{g}_{m}}\:\frac{d{g}_{m}}{dt}={P}_{H}-\:{P}_{o}$$

- The arc heat power was assumed to be equal to the electrical power generated from the arc (v $\:\times\:\:$i), where v is the arc voltage and i is the arc current. Then (2) was substituted (2) in (3) to get (4).

4 $$\:\frac{{P}_{o}\:\tau\:}{{g}_{m}}\:\frac{d{g}_{m}}{dt}=(v\:\times\:i)-\:{P}_{o}$$

- By rearranging Eq. (4) while considering that conductance $\:{g}_{m}=v/i$, we get the final form in (5).

5 $$\:\frac{d{g}_{m}}{dt}=\frac{1}{\tau\:}\left(\frac{{i}^{2}}{\:{P}_{o}}-\:{g}_{m}\right)$$

The previous formula is used for determine the arc conductance and subsequently its resistive effect. This part is associated with a contact model. When the trip signal is received, each stage model is activated at the corresponding time till reaching the opened state. The MODELS tool available on ATP/EMTP inn addition to TACS controlled resistor and ideal switch were all used in accordance to the four stage steps previously described in aim to model the SF_6_ interrupter. The MODELS tool implements the mathematical computation of the arc conductance based on the measured electrical data from the system.

### Modeling of the shunt branches

The L-C commutating branch is modeled using L, C components that are connected in parallel with the black box model^20^. The values of Land C will be varied within the simulation section to define their most suitable values. The MOV was modeled using its assigned model upon ATP that is characterized by non-linear current-voltage characteristic allowing it to respond to overvoltage generated in power systems^20^. This element is used to store the excess energy generated from the fault incident.

## Simulation results

The testing is process will be divided into phases to fulfill the aims required from the simulation part. The aims were numerated earlier in second section; these were:

1. To evaluate the performance of UHAD-CB in HVAC system.
2. Compare its perform to conventional HVAC-CB that were used with higher settings to ensure that the reduction in settings for UHAD-CB will not affect its performance.
3. Evaluate the performance of UHAD-CB in HVAC system.
4. Investigate the most suitable values for shunt L-C branch for both systems.

To satisfy these aims, the following phases are followed in the testing process.

- **Phase I**: Test conventional HVAC-CB with higher settings within HVAC system previously defined. The results from this phase are to be used in the next phase.
- **Phase II**: Test UHAD-CB with lower settings than those used in phase I. The results from this phase are used in three aims as follows:
- They are compared to the results from previous phase to fulfill aim number 2.
- They will show the performance of UHAD-CB with HVAC system that fulfills aim number 1.
- Through investigation, the most appropriate L and C values will be defined for HVAC system partially fulfilling aim number 4.
- **Phase III**: the UHAD-CB is tested in HVDC system to fulfill aims 3 and 4. Thus by the end of this phase all aims are reached and conclusion could be driven.

### Phase I: conventional HVAC-CB

This phase examines the performance of conventional HVAC-CB to be latter compared with UHAD-CB. The testing system will be the HVAC transmission line system earlier described in previous section. The settings of the HVAC-CB will include cooling power for the arc provided by the CB. The mechanism for cooling the arc will depend on the features of SF6 interrupter that requires additional cost to reach higher cooling powers. The range of cooling powers selected in this phase for the HVAC-CB were from 100 MW up to 140 MW that were based on typical values used by researchers^36^. For each one, two scenarios were assumed. The first id the occurrence of a three phase 0.01-ohm fault across the HVAC line at 5 ms.

**Fig. 3:**
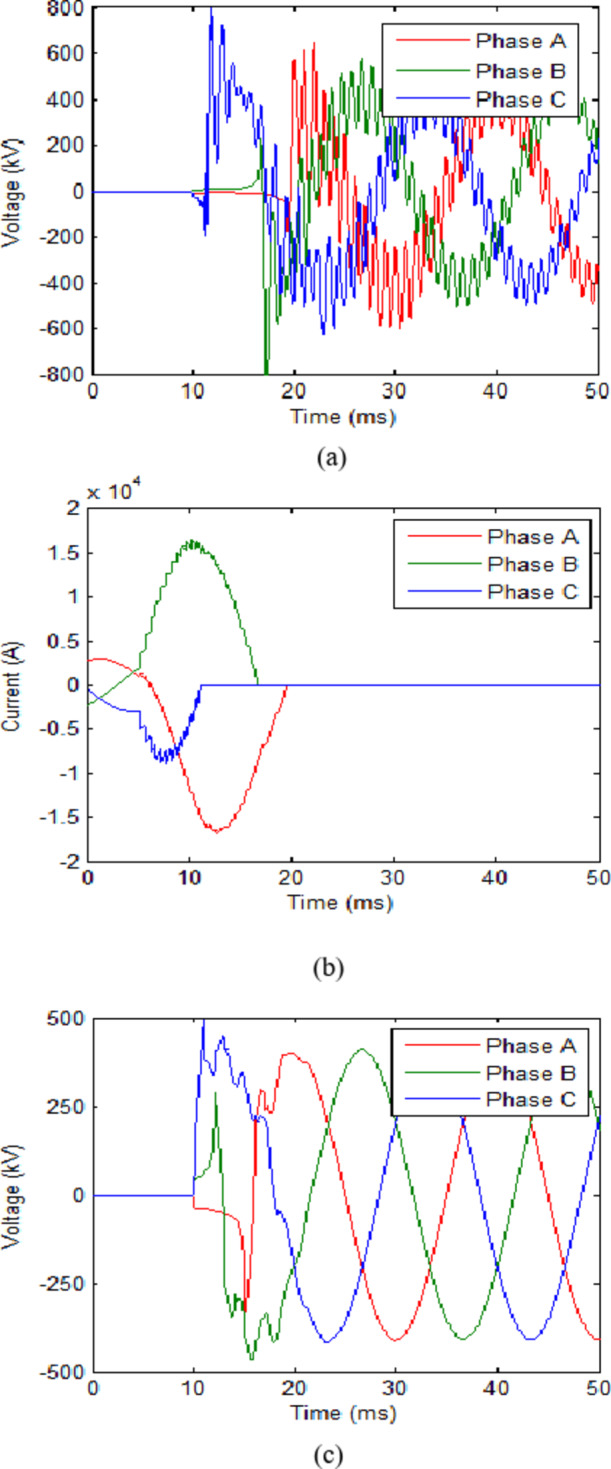
HVAC-CB with 100 MW cooling power (**a**) Voltage across CB during fault scenario and (**b**) Current through CB during fault scenario. (**c**) Voltage across CB during no-fault scenario.

**Fig. 4:**
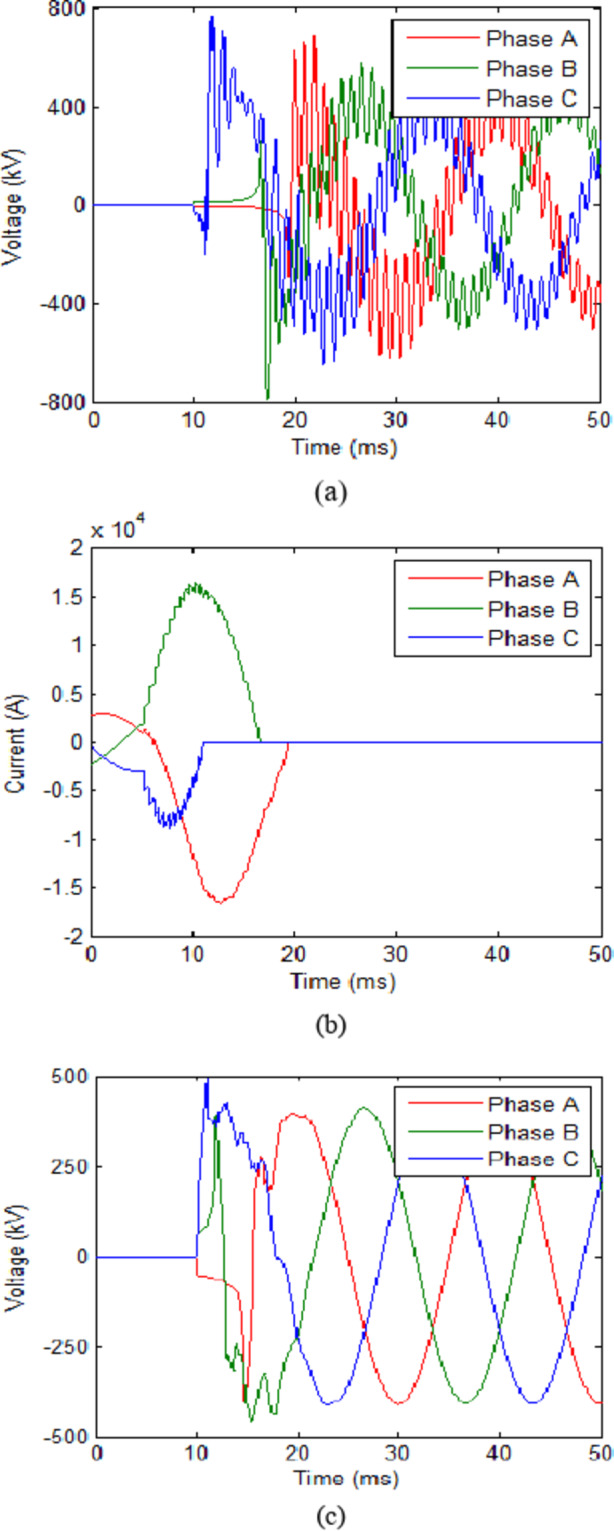
HVAC-CB with 140 MW cooling power (**a**) Voltage across CB during fault scenario and (**b**) Current through CB during fault scenario (**c**) Voltage across CB during no-fault scenario.

The trip signal was assumed to reach the CB at 10 ms. While the second scenario assumes that no faults have occurred and CB receives an opening signal at 10 ms as part of a regular switching process. The maximum value reached for the transient recovery voltage (TRV) was recorded. The results for both scenarios in each case are presented in Table 2. The voltage across the CB and current flowing through it for cooling powers of 100 MW and 140 MW are shown in Figs. 3 and 4 respectively. The results show that during fault scenarios, the TRV were in range of 800 kV that were slightly reduced by increasing the cooling power. While for non-fault scenarios, the TRV was almost the same in all cases in range of 490 kV. These results will be compared to those of UHAD-CB in the next phase.

**Table 2:** Results of phase I.

Cooling Power (MW)	Maximum TRV recorded (kV)	
	With fault	Without Fault
100	817.8	494.8
110	811.9	494.8
120	805.1	494.8
130	798.14	494.8
140	790.5	494.8

### Phase II: UHAD-CB in HVAC System

This phase installs the UHAD-CB within the HVAC testing transmission line. Two aims are intended to be addressed in this phase. To compare the performance of lower settings UHAD-CB with previous results of HVAC-CB and to investigate the proper selection for L and C values. For such reason, two main steps are done within this phase:

- The first step is to evaluate the performance of the UHAD-CB.
- While the second step will deal with proper setting of L-C values.

**Fig. 5:**
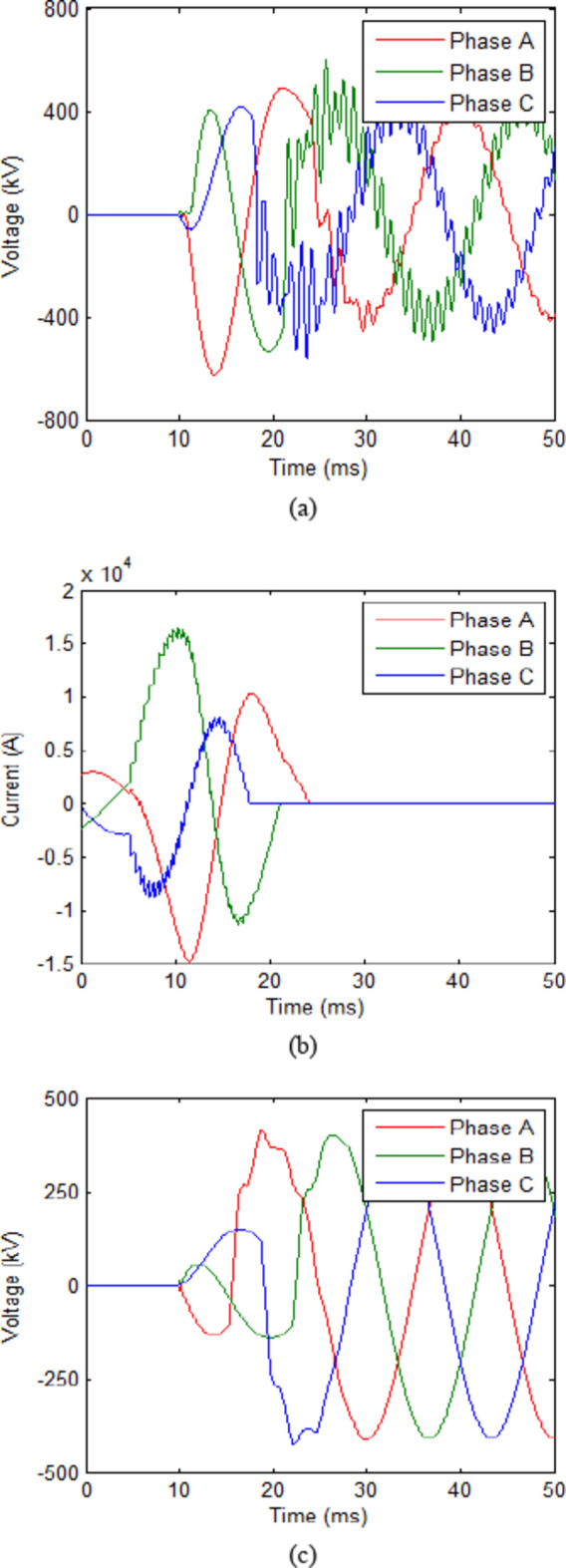
UHAD-CB with 100 MW cooling power, L = 0.2 mH and C = 40 µF in HVAC system (**a**) Voltage across CB during fault scenario and (**b**) Current through CB during fault scenario (**c**) Voltage across CB during no-fault scenario.

**Fig. 6:**
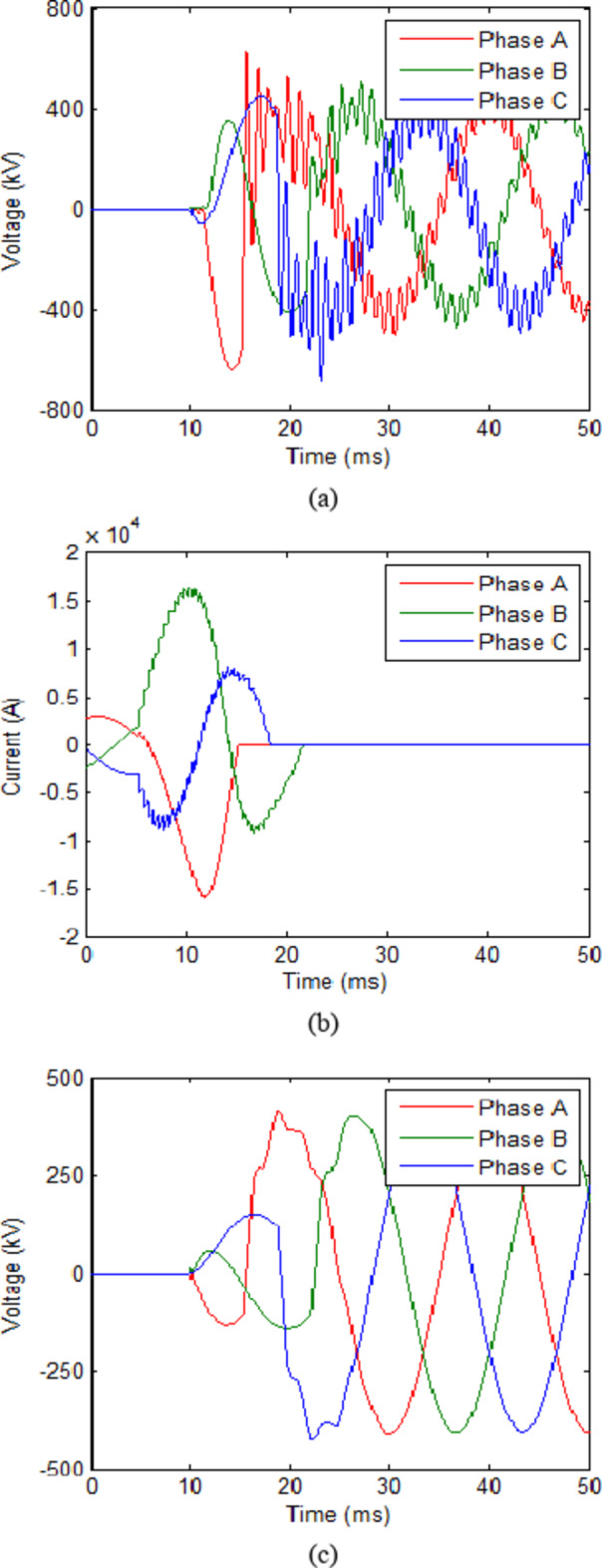
UHAD-CB with 80 MW cooling power, L = 0.2 mH and C = 40 µF in HVAC system (**a**) Voltage across CB during fault scenario and (**b**) Current through CB during fault scenario (**c**) Voltage across CB during no-fault scenario.

For the first step, the L and C are set to 0.2 mH and 40 µF respectively. These were selected as an initial value that are subject to change based on the TRV results. The initial values of L and C are loosely based on those chosen for CBs in HVDC systems in^20,22^. However, since these researches are directed for HVDC systems only, their values were considered as an approximation point to start an initial value for L and C. The cooling power of the UHAD-CB was set to 100 MW which the was the minimal power used in previous phase. The results for this case are shown in Table 3. The results show that the maximum TRV reached 624.6 kV and 424.7 kV for fault and no-fault scenarios respectively. The cooling power was furtherly reduced to 80 MW as shown in second row of Table 3. The results show that the UHAD-CB at 80 MW had a 686.7 kV TRV which is much lower the HVAC-CB at 140 MW. Hence, the results show that the UHAD-CB can effectively operate with a lower cooling power down to 80 MW would still provide a better performance than HVAC-CB at 140 MW and hence, any additional cost for the shunt branches are compensated by the use of lower cooling power SF_6_ interrupter. The voltage and current across CB for 100 MW and 80 MW cases are shown in Figs. 5 and 6 respectively.

**Fig. 7:**
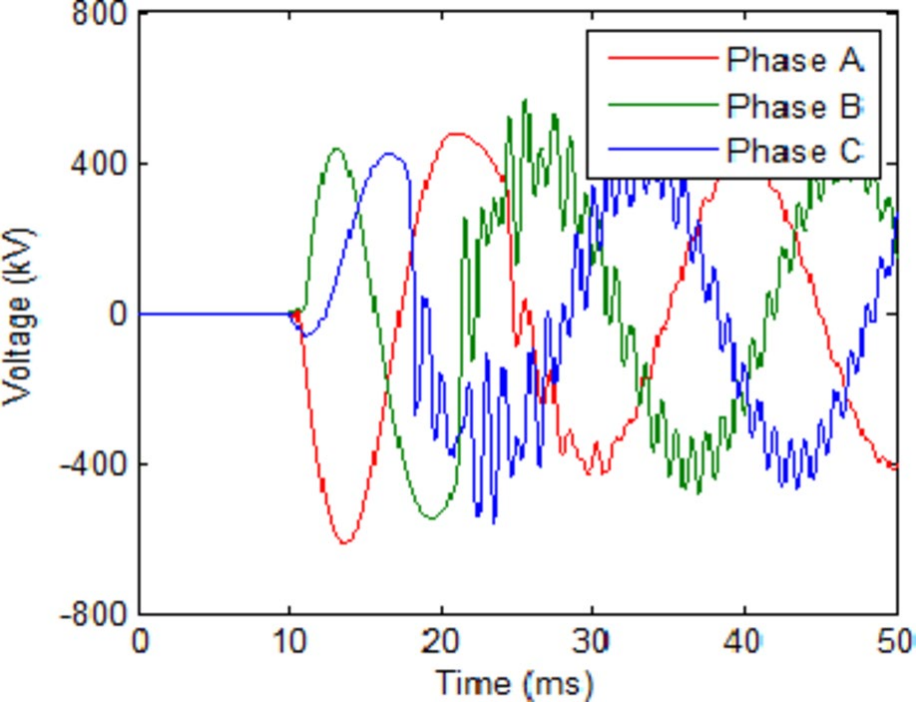
Voltage across UHAD-CB with 80 MW cooling power in HVAC system during fault scenario with L = 0.1 mH C = 40 µF.

**Fig. 8:**
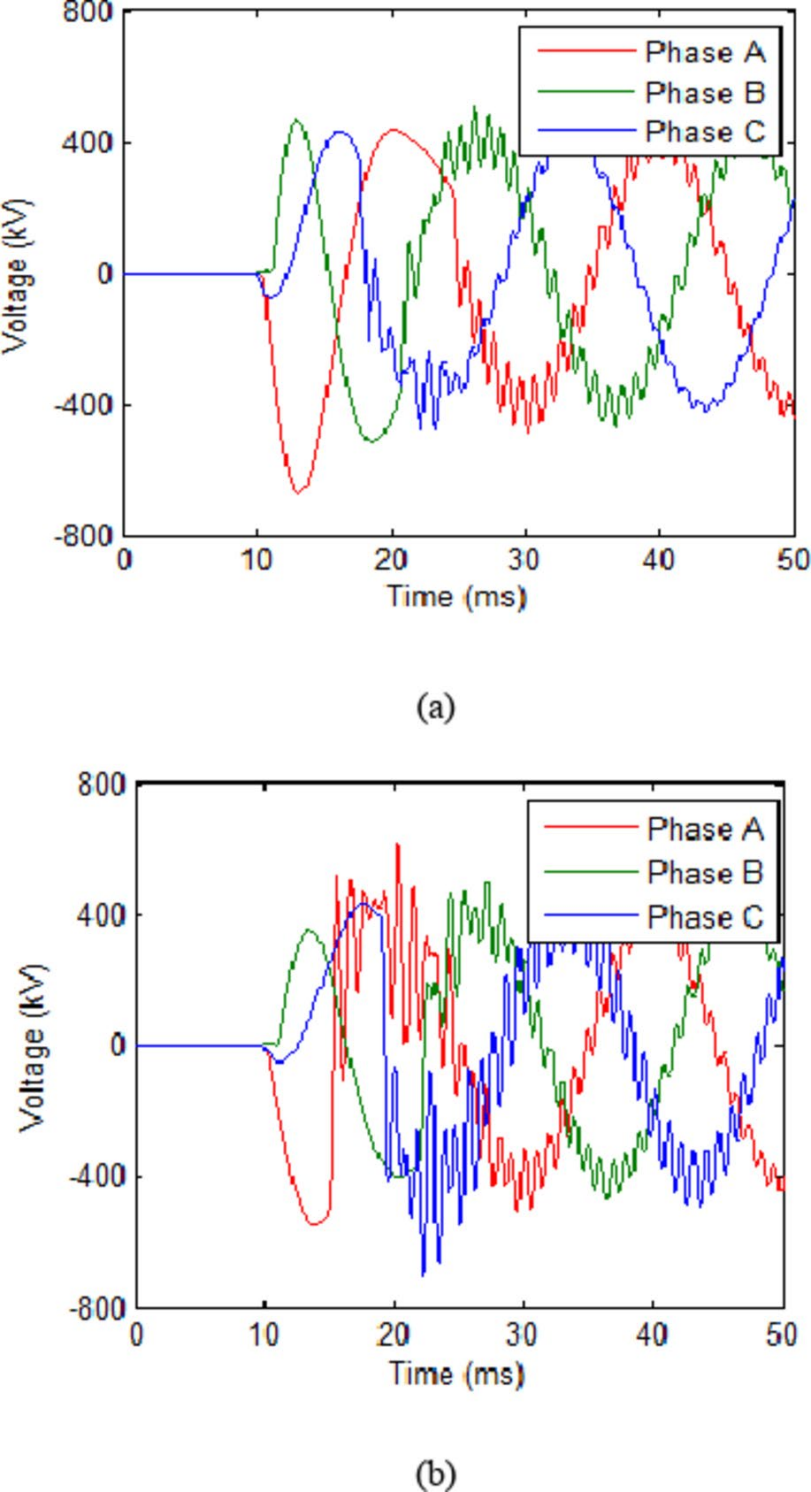
Voltage across UHAD-CB with 80 MW cooling power in HVAC system during fault scenario with L = 0.1 mH (a) C = 30 µF (b) (a) C = 60 µF.

The second step of this phase examines the suitable values for UHAD-CB while continuing to use 80 MW cooling power design. During this step, the L will be firstly varied to reach most acceptable TRV while keeping the C constant as seen in the third and fourth rows of Table 3. Varying the value of L from 0.3 mH to 0.1 mH showed the L = 0.1 mH had the least TRV of 613.6 kV. The no-fault scenario TRV was almost constant in all cases. Keeping L constant at 0.1 mH and changing the value of C is done in rows 5 and 6 of Table 3. Increasing the capacitance to 50 µF showed a reduction in TRV to 587 kV. The voltage for cases in fourth and fifth rows of Table 3 are shown in Figs. 7 and 8(a) respectively. Further increase in capacitance to 60 µF and 70 µF lead to increase in TRV which reached 703 kV and 691.9 V respectively. This is explainable based on Fig. 8(b) which shows the TRV across the CB in the case with C = 60 µF. It is observable that the highest TRV reached was for phase C at approximately 22 ms. That sudden increase is due to the high frequency oscillations associated with the L-C circuit. Such oscillations were minimal in previous cases as shown in Figs. 7 and 8 (a). As in these figures the highest TRV was right after the opening instant of the CB as seen in phase A in these figures. Meanwhile the associated high frequency oscillations in these figures that is shown starting from time of 20 ms were smaller than the firstly recorded TRV. Therefore, the results indicated that the L-C are capable of reducing the TRV but the injected high frequency oscillations from L-C could in increase the TRV at certain ranges and hence, the prior simulation and testing are required as done in this paper. Finally, for this system and based on the testing results and the results of Table 3, it is shown that minimum TRV was reached at 50 µF and hence, the most suitable value for C will be 50 µF for this system. Finally, it is recommended to use variable L and C that could varied if the CB is to transferred to another system or if optimum values of L and C were varied for any changes in the system. Thus, allowing more flexible design of UHAD-CB.

It is also noted that L-C did not affect the steady state voltage waveform nor the power transfer of the line. As for HVAC-CB without L-C, the steady state value was almost 408.2 kV and the same value was recorded for UHAD-CB with L-C connected. Also, the presence of L-C did not affect the power transfer of the line as it could be concluded by comparing the value of the steady state currents from Fig. 4(b), 5(b). In the first 5 ms of Fig. 4(b), the steady state current is exactly the same as the steady state current in the same time range in Fig. 5(b) where L-C was added. The previous results are logical as the L-C is connected in parallel with the SF6 interrupter as shown in Fig. 1. This means when the CB completely closes to reach steady state, the L-C will be short circuited and will not affect the steady state parameters. It will only affect the transient parameters during which the CB is changing its state.

Another aspect for improving the TRV response for UHAD-CB in HVAC systems is including also MOV within the CB. This was examined in the last three cases of Table 3. These cases include MOV in addition to L-C branches with values that were tested earlier. It is observable that for L-C branch with values of 0.1 mH and 30 µF respectively, the addition of MOV reduced the TRV from 671.1 kV in row number to 644.5 in row number 9. The same applies for C = 40 µF and 50 µF. However, the amount of reduction is to be compared with the added price of MOV. This is dependent on each utility or consumer needs and priorities. By the end of the second, the second phase had accomplished its aims numbers 2 and 4.

**Table 3:** Results of phase II.

No.	Cooling Power (MW)	UHAD-CB Parameters		MOV	Maximum TRV recorded (kV)	
		L (mH)	C (µF)		With fault	Without Fault
1	100	0.2	40	-	624.6	424.7
2	80	0.2	40	-	686.7	424.7
3	80	0.3	40	-	747.3	424.7
4	80	0.1	40	-	613.6	424.7
5	80	0.1	30	-	671.1	424.7
6	80	0.1	50	-	587.2	429.77
7	80	0.1	60	-	703.1	431.2
8	80	0.1	70	-	691.9	431.2
9	80	0.1	30	used	644.5	424.7
10	80	0.1	40	used	601.3	424.7
11	80	0.1	50	used	568.4	429.6

### Phase III: UHAD-CB in HVDC system

The final phase will test the UHAD-CB within the HVDC system. This phase tends to fulfil aims number 3 and 4. This includes ensuring reliable performance of UHAD-CB within HVDC system and investigating the L-C range of suitable values within this system. An added task will be combining the range for L-C values from pervious phase within phase to define the intersecting range for L-C values that satisfies both systems. It should be mentioned that in this phase, an MOV is installed in its assigned compartment within the UHAD-CB. The parameters for the MOV are as mentioned in the second section. The initial L-C values were 0.2 mH and 30 µF which resulted in a 733 kV peak for transient voltage as presented in Table 4. Increasing the capacitance to 40 µF and 50 µF reduced the TRV to 720 and 693 respectively. Thus, the 50 µF is considered the reasonable selection for the value of C.

**Fig. 9:**
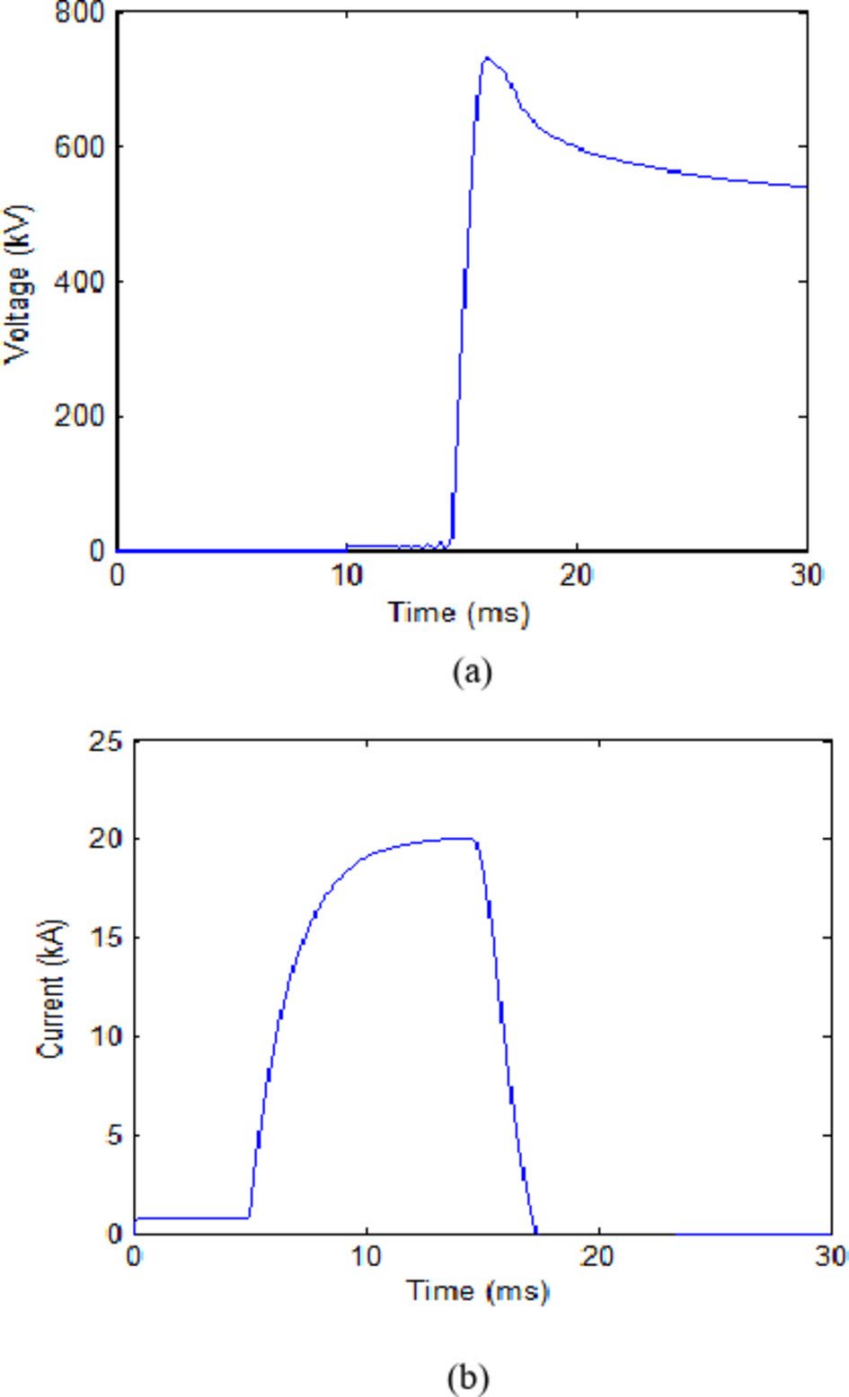
Results of UHAD-CB with 80 MW cooling power in HVDC system during fault scenario with L = 0.2 mH C = 30 µF (**a**) Voltage across CB (**b**) Current through CB.

**Fig. 10:**
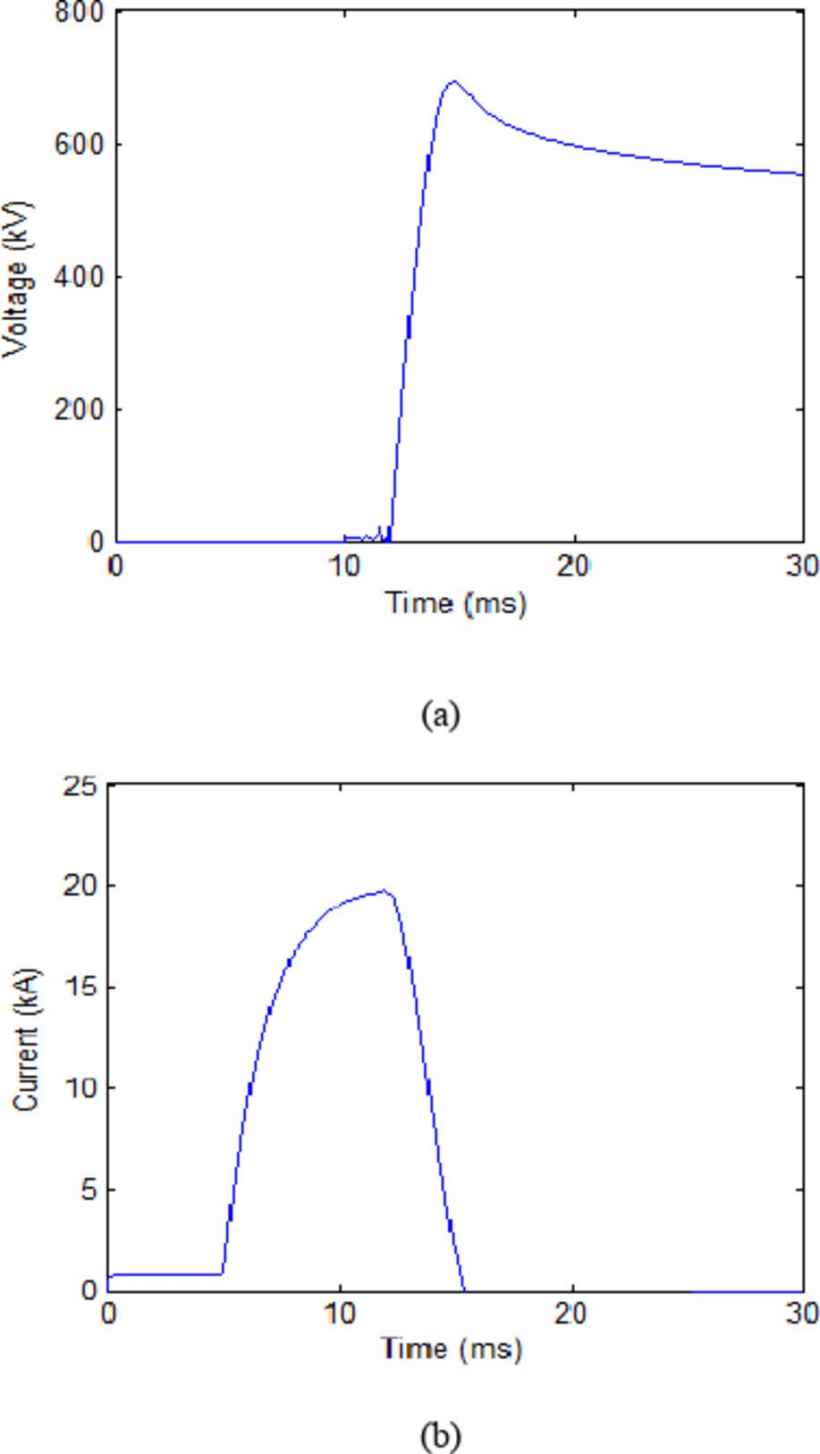
Results of UHAD-CB with 80 MW cooling power in HVDC system during fault scenario with L = 0.1 mH C = 50 µF (**a**) Voltage across CB (**b**) Current through CB.

The following step fixes the value of C and varies the value of L, such that L is and decreased to 0.1 mH and later increased to 0.3 mH. Those changes resulted in TRVs of peaks of 692.3 kV and 694.71 kV respectively. Thus the optimum L-C value from the HVDC system point of view will be 0.1 mH and 50 µF. These values are the same as those selected for HVAC-system. The previous results are not general results but are specified for the selected testing systems. If these systems are changed, the optimum value of L and C will change. The results for initial case (first row in Table 4) and the selected case (fourth row of Table 4) are shown in Figs. 9 and 10 respectively.

**Table 4:** Results of Phase III.

Cooling Power (MW)	UHAD-CB Parameters		Maximum TRV recorded (kV)
	L (mH)	C (µF)	
80	0.2	30	733.04
80	0.2	40	720.01
80	0.2	50	693.55
80	0.1	50	692.38
80	0.3	50	694.71

### Analysis of the results

**Fig. 11:**
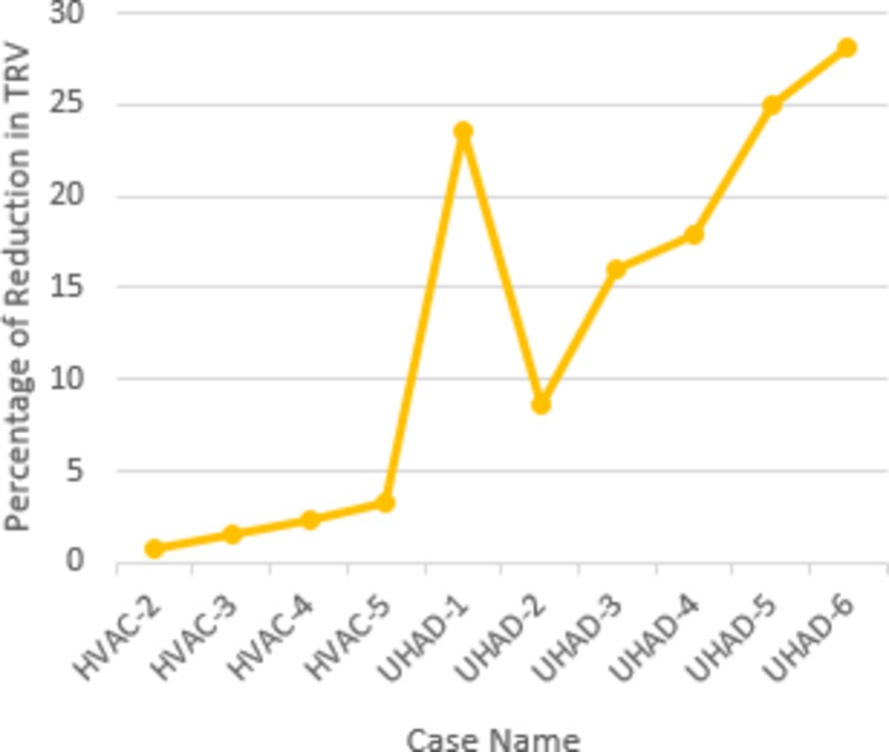
Percentage of reduction in TRV for HVAC-CB and UHAD-CB.

The results for the simulation had proven the trustable performance of UHAD-CB in both HVDC and HVAC systems. The assigned aims for that section were satisfied. However, the performance of UHAD-CB was not evaluated completely. This section tends to provide evaluative analysis for the performance of UHAD-CB. To perform this task, an index is used to measure the performance of CB in terms to its ability to suppress the TRV. This index will consider the TRV reached by conventional HVAC-CB with 100 MW cooling power as reference value. This value was considered in this paper as the target is to evaluate the UHAD-CB with respect to HVAC-CB, hence one of the results of HVAC-CB was selected. Since the 100 MW cooling power was the first one to be tested within HVAC-CB, then it was selected as arbitrary reference. The index will compare the resulting TRV of a certain case with respect to the mentioned reference by computing the difference between the TRV of the selected case and the TRV from reference case and divide that difference by the TRV from reference case. The index will be termed as percentage of reduction in TRV. That percentage was calculated for results from phase I and II that were presented in Tables 2 and 3 respectively. The results are presented in Table 5. It should be mentioned the results of UHAD-CB in HVDC system don’t need comparison as the structure employed for the UHAD-CB is the same of HVDC-CB. The cases within Table 5 were number using type of CB as initials and numbering order to be using within graphical representation in Fig. 11.

**Table 5:** Evaluation for UHAD-CB.

Type of CB	Case no.	Cooling Power (MW)	UHAD-CB Parameters		Percentage of reduction in TRV
			L (mH)	C (µF)	
HVAC-CB	HVAC-1	100	-	-	Reference case
	HVAC-2	110	-	-	0.72
	HVAC-3	120	-	-	1.55
	HVAC-4	130	-	-	2.40
	HVAC-5	140	-	-	3.34
UHAD-CB	UHAD-1	100	0.2	40	23.62
	UHAD-2	80	0.3	40	8.62
	UHAD-3	80	0.2	40	16.03
	UHAD-4	80	0.1	30	17.94
	UHAD-5	80	0.1	40	24.97
	UHAD-6	80	0.1	50	28.20

The results from both the figure and the table show the increased ability for UHAD-CB in reducing TRVs in comparison with conventional HVAC-CB. This was shown from the percentage of reduction in TRV that reached up to 28.2% for UHAD-CB while for HVAC-CB reached only 3.34%. This enhanced performance is attributed to the contribution of the shunt branches. Therefore, it could be summarized that in addition to the main aim of this paper which introducing a universal structure frame for high voltage CBs for both HVDC and HVAC system, the proposed UHAD-CB proved higher performance with respect to conventional HVAC-CB. A notable drawback is still observable which is increasing the arcing time of UHAD-CB. This is observed in Fig. 5(b) and 6(b) in which the arc was extinguished at 25 ms while the fault was initiated at 5 ms so the arcing time is range of approximately 20 ms. While for HVAC-CB, the arcing time was in range of 15 ms. That increase in arcing time is expected as UHAD intended to reduce the TRV and this means that the amount of energy associated with the arc was redistributed in extended time range to reduce the TRV. Hence, the arcing time increased. Although this is a drawback but that should be compared to the gained advantages from the UHAD-CB, which is higher performance in terms of reducing the TRV and allowing universal structure for HVDC and HVAC CBs that will allow wider mass production of the CBs and reduce the overall cost for the manufactures and the utilities.

To further specify about the economic impact of using UHAD-CB with lower cooling power as 80 MW instead of HVAC-CB with higher cooling power of 140 MW as presented in Table 5, analysis is presented. Even though that exact prices are not available for data related to manufactures, a price range is provided for high voltage SF6 circuit breaker in^37^. That range is averaged at 100, 000 USD. The exact price will depend on manufacturer. However, this paper focuses not on the exact price but the approximated increases in price for 80 MW to 140 MW cooling power of CB. Such increase is reflected in the interrupting current which would range in 10,000 USD increase in price between the two previously mentioned CBs. That means that UHAD-CB is expected to be cheaper by 10,000 USD than its counterpart of HVAC-CB without considering the added price for L-C branch. The added price of L-C branch for UHAD-CB will not exceed 1000 USD according to^38^. So finally, the UHAD-CB with L-C branch is expected to have a 9000 USD cheaper price than HVAC-CB. The previous analysis will not be able to consider the effect of mass production for HVAC and HVDC systems which will further reduce the price UHAD-CB.

## Conclusions

The high voltage transmission field had extended within two main domains the HVDC and HVAC systems. Both intended for different circumstances. That mandated a need for CBs for each system that increased the manufacturing burden and bill cost for the power system utilities. A solution for this was presented in this paper by proposing a concept for a universal high voltage circuit breaker (UHAD-CB) that could be used in both systems with limited modifications. The UHAD-CB is based on the structure for the HVDC-CB that utilizes SF_6_ interrupter and L-C shunt branches to inject an artificial zero crossing.

The UHAD-CB was tested upon two testing systems; HVAC transmission system and HVDC transmission system. The results showed the following:

1. UHAD-CB had proven to perform reliably in both HVDC and HVAC systems.
2. The UHAD-CB reduced TRV in comparison to HVAC-CB by a range reaching 28%.
3. The added cost for shunt branches is compensated for using lower cooling power SF_6_ interrupter while maintaining a reliable performance.
4. The values of the most suitable L and C for the selected systems were 0.1 mH and 50 µF. However, these values are based on system parameters and designer’s choice.

The previous conclusions show that the proposed UHAD-CB is a feasible concept that might be furtherly investigated through extensive research to reach a final form its structure. This would allow manufactures to maintain a single production line for HVCBs that would reflect in faster production rates, lower overall costs and benefits for the entire industry.

## Data Availability

The data that support the findings of this study are available from the corresponding author upon reasonable request.
